# Correction: Hexameric and pentameric complexes of the ExbBD energizer in the Ton system

**DOI:** 10.7554/eLife.37833

**Published:** 2018-05-04

**Authors:** Koji Yonekura, Saori Maki-Yonekura, Rei Matsuoka, Yoshiki Yamashita, Hirofumi Shimizu, Maiko Tanaka, Fumie Iwabuki

Maki-Yonekura S, Matsuoka R, Yamashita Y, Shimizu H, Tanaka M, Iwabuki F, Yonekura K. 2018. Hexameric and pentameric complexes of the ExbBD energizer in the Ton system. *eLife* **7**:e35419. doi: 10.7554/eLife.35419.

Published 17, April 2018

We measured the channel current at pH 7.5 and pH 4.5 with a voltage of ± 50 mV in (A). Macroscopic current were measured upon voltage steps from -50 to 50 mV in (C) and (D).

In the originally published Figure 3CD and Figure 3A legend we incorrectly miswrote the voltage in V instead of mV. We have now corrected these typographical errors. The correction does not affect the results and conclusions of the original article.

The corrected Figure 3 is shown here:

**Figure:**
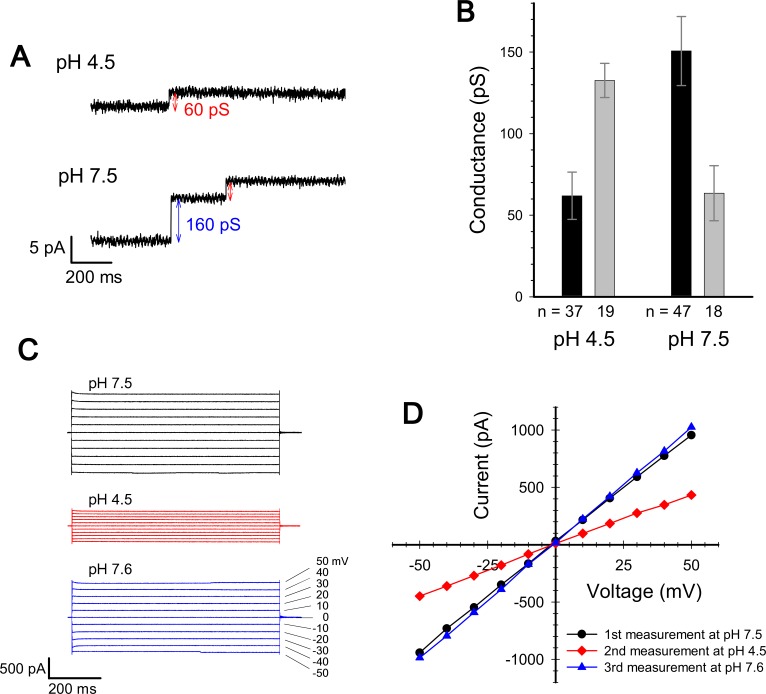


The originally published Figure 3 is also shown for reference:

**Figure:**
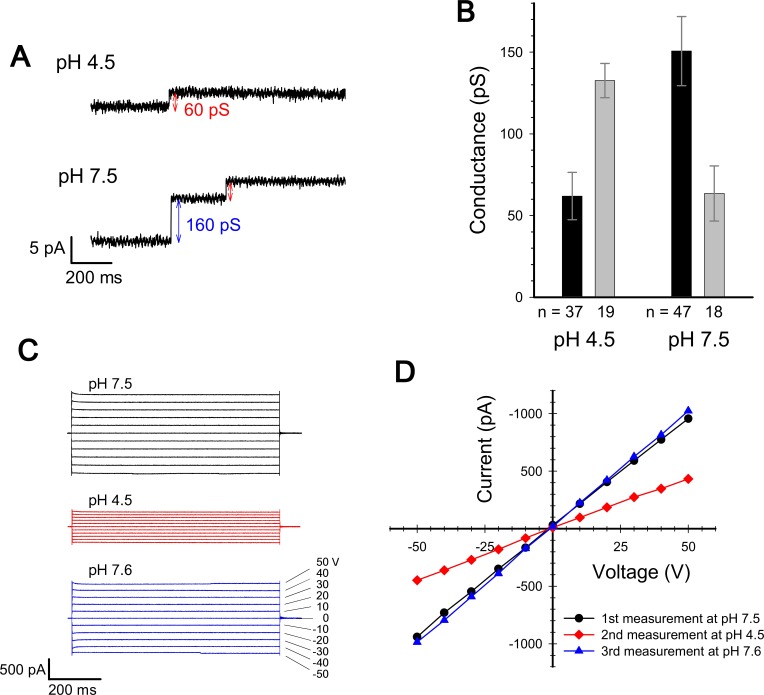


The corrected Figure 3A legend text is shown below:

(A) Typical charts of channel current at pH 7.5 and pH 4.5 with a voltage of ± 50 mV.

The article has been corrected accordingly.

