# Supplementary material for: Hexameric and pentameric complexes of the ExbBD energizer in the Ton system
Source: eLife. 2018 Apr 17;7:e35419. doi: 10.7554/eLife.35419 (PMC5903867; doi:10.7554/eLife.35419)
Supplement: Supplementary file 1. — Data collection and image analysis statistics for single particle cryo-EM. Table S3. Refinement statistics of atomic models against the cryo-EM maps [file elife-35419-supp1.docx]

**Supplemental Information**

**Hexameric and pentameric complexes of**

**the ExbBD energizer in the Ton system**

Saori Maki-Yonekura^1^, Rei Matsuoka^1^,Yoshiki Yamashita^1^,

Hirofumi Shimizu^2^, Maiko Tanaka^1^, Fumie Iwabuki^1^, and Koji Yonekura^1, *^

* Correspondence: yone@spring8.or.jp

**Table S1**

**Data collection and refinement statistics of the ExbB and ExbD crystals grown at pH 9**

**Data collection**

Space group *P*2_1_ *P*1 *P*1 ^*^

Cell dimensions

*a*, *b*, *c* (Å) 121.5, 106.3, 163.4 71.4, 71.9, 205.9 71.4, 71.4, 212.7

*α*, *β*, *γ* (º) 90, 111.75, 90 93.6, 90.4, 119.9 90.5, 91.1, 120.1

Resolution (Å) 50.18 – 2.84 47.88 – 3.00 47.23 – 3.01

(2.94 – 2.84) (3.08 – 3.00) (3.09 – 3.01)

Completeness (%) 99.3 (99.0) 94.0 (89.8) 92.9 (88.2)

CC1/2 (%) 99.2 (75.1) 98.4 (18.1) 89.8 (10.4)

*R*_merge_ 0.11 (0.500) 0.173 (1.64) 0.354 (2.33)

*R*_meas_ 0.155 (0.706) 0.245(2.32) 0.50 (3.29)

*I/σ* 6.67 (1.71) 4.21 (0.52) 3.67 (0.90)

Number of crystals 1 1 1

**MR solution with an initial ExbB hexamer structure**

LLG ^†^ 1199 1316 1667

TFZ ^‡^ 17.5 24.4 24.7

**MR solution with the refined ExbB hexamer structure**

LLG ^†^ - 4410 4669

TFZ ^‡^ - 49.3 48.0

**Refinement**

Resolution (Å) 50.18 – 2.84 (2.91 – 2.84)

Completeness (%) 99.3 (99.4)

*R*_work_ 0.245 (0.242)

*R*_free_ 0.288 (0.297)

R.m.s deviations

Bond lengths (Å) 0.01

Bond angles (º) 1.11

Ramachandran plot (%)

Favored 99.58

Allowed 0.42

Outliers 0

^*^ The histidine tag was cleaved off.

^†^ Log-likelihood gain (McCoy et al., 2007). LLG should be positive and high for the likely solution.

^‡^ Translation function Z score (McCoy et al., 2007). TFZ > 8 indicates the likely solution.

**Table S2**

**Data collection and image analysis statistics for single particle cryo-EM**

| Sample pH | 8.0 | 5.4 | 8.0 ^*^ | 7.0 | 9.0 | | |
| --- | --- | --- | --- | --- | --- | --- | --- |
| Microscope | Polara ( UCSF ) | Krios  ( Diamond ) | | Arctica  ( RIKEN ) | | | |
| Imaging device | K2 Summit | Quantum  K2 Summit | | K2 Summit | | | |
| Voltage (kV) | 300 | 300 | | 200 | | | |
| Defocus range (μm) | 0.6 － 3.1 | 0.6 － 3.1 | 0.6 － 2.9 | 0.5 － 4.9 | | | 0.5 － 3.0 |
| Exposure time (s) | 20 | 6 | 10 | 6.4 | | | |
| Dose rate (e^-^ / pixel / s) | 6.0 | 6.5 | 5 | 7.5 | | | |
| Pixel size (Å) | 1.22 | 1.06 | 1.06 | 0.98 | | | |
| Pentamers / hexamers  in 2D averages projected along the channel axis | 10,422 /  30,323 | 24,856 /  8,324 | 8,042 /  22,541 | 5,296 /  4,562 | | Not Detected / 2,704 | |
| Particles processed for 3D reconstruction | 276,526 | 122,908 | － | － | | － | |
| Particles refined | 38,323 | 22,243 | － | － | | － | |
| Resolution (unmasked) in Å | 7.36 | 7.16 | － | － | | － | |
| Resolution (masked)  in Å | 6.69 | 7.11 | － | － | | － | |
| Map sharpening  B-factor (Å^2^) | -192 | -50 ^†^ | － | － | | － | |

^*^ The histidine tag was cleaved off.

^†^ Selected manually to avoid over amplification of detergent densities.

**Table S3**

**Refinement statistics of atomic models against the cryo-EM maps**

Model ExbB_6_D_3TM_ ExbB_5_D_1TM_

CC coefficient 0.665 0.665

All atom clash score* 5.57 17.05

R.m.s deviations

Bond lengths (Å) 0.01 0.01

Bond angles (º) 0.95 1.13

Ramachandran plot (%)

Favored: 98.31 94.48

Allowed: 1.25 5.52

Outliers: 0.44 0.00

^*^ Calculated as 1,000 × number of bad overlaps / number of atoms (Adams et al., 2010).
